# AT1 Receptor Mediated Hypertensive Response to Ang II in the Nucleus Tractus Solitarii of Normotensive Rats Involves NO Dependent Local GABA Release

**DOI:** 10.3389/fphar.2019.00460

**Published:** 2019-05-03

**Authors:** Laura Légat, Ilse Smolders, Alain G. Dupont

**Affiliations:** ^1^Department of Pharmaceutical Chemistry, Drug Analysis and Drug Information, Research Group Experimental Pharmacology, Center for Neurosciences, Vrije Universiteit Brussel, Brussels, Belgium; ^2^Cardiovascular Center, Universitair Ziekenhuis Brussel, Brussels, Belgium; ^3^Department of Clinical Pharmacology and Clinical Pharmacy, Universitair Ziekenhuis Brussel, Brussels, Belgium

**Keywords:** angiotensin II, gamma-aminobutyric acid (GABA), mean arterial pressure, nucleus tractus solitarii, nitric oxide

## Abstract

**Aim:**

It is well-established that angiotensin II exerts a dampening effect on the baroreflex within the nucleus tractus solitarii (NTS), the principal brainstem site for termination of baroreceptor afferents and which is densely populated with gamma-aminobutyric acid (GABA)ergic neurons and nerve terminals. The present study was designed to investigate whether local release of GABA is involved in the effects mediated by local angiotensin II within the NTS.

**Methods:**

*In vivo* microdialysis was used for measurement of extracellular glutamate and GABA levels and for infusion of angiotensin II within the NTS of conscious normotensive Wistar rats. The mean arterial pressure (MAP) and heart rate response to local infusion of angiotensin II were subsequently monitored with a pressure transducer under anesthesia. The angiotensin II type 1 receptor (AT1R) antagonist, candesartan, was used to assess whether responses were AT1R dependent and the nitric oxide (NO) synthase inhibitor, *N*(ω)-nitro-L-arginine methyl ester (L-NAME), was used to assess the involvement of NO in the evoked responses by infusion of angiotensin II. The MAP and heart rate responses were monitored with a pressure transducer.

**Results:**

Local infusion into the NTS of angiotensin II induced a significant to ninefold significantly increase in extracellular GABA levels; as well as MAP was increased by 15 mmHg. These responses were both abolished by co-infusion of either, the angiotensin II type 1 receptor antagonist, candesartan, or the NO synthase inhibitor, L-NAME, demonstrating that the effect is not only AT1R dependent but also NO dependent. The pressor response to angiotensin II was reversed by co-infusion with the GABA_A_ receptor antagonist, bicuculline. Local blockade of NO synthase decreased both, GABA and glutamate concentrations.

**Conclusion:**

Our results suggest that the AT1R mediated hypertensive response to angiotensin II within the NTS in normotensive rats is GABA and NO dependent. Nitric oxide produced within the NTS tonically potentiates local GABA and glutamate release.

## Introduction

The central nervous system network that regulates the level of sympathetic tone and, hence, blood pressure, is located in the brainstem and consists mainly of the rostral ventrolateral medulla (RVLM), the paraventricular nucleus (PVN) of the hypothalamus and the nucleus tractus solitarii (NTS) (Guyenet, 2006). It is well-established that the RVLM, the so-called brainstem ‘pressor area,’ is a key site for the regulation of sympathetic tone and blood pressure (Dupont and Brouwers, 2010). The major source of excitatory sympathetic drive from the RVLM are spinally projecting glutamatergic neurons which receive tonic excitatory signals from other brain areas, including the PVN, and inhibitory signals from the caudal ventrolateral medulla (CVLM) and indirectly from the NTS (Guyenet, 2006). The NTS plays an important role in central cardiovascular regulation and is the principal site for the termination of baroreceptor afferent fibers mediating the inhibitory actions of baroreceptors in the arterial wall on sympathetic outflow (Paton and Kasparov, 1999; Guyenet, 2006). Stimulation of mechanosensitive baroreceptors by distension of the arterial wall following blood pressure elevation results in activation of glutamatergic neurons located in the NTS. The dampening effect on the barosensitive neurons of RVLM is mediated by the gamma-aminobutyric acid (GABA)-containing neurons of the CVLM, indicating a crucial role of GABA-mediated input from the CVLM for the baroreflex. The second-order NTS neurons indeed project to and activate GABAergic neurons within the CVLM, which have an inhibitory influence on RVLM neurons. Hence, baroreceptor mediated activation of NTS neurons indirectly inhibits the RVLM through activation of GABAergic neurons of the CVLM, ultimately resulting in a reduced sympathetic outflow with blood pressure and heart rate (HR) reduction (Guyenet, 2006).

The role of the brain renin–angiotensin system (RAS), and in particular of the angiotensin II type 1 receptor (AT1R), in the regulation of blood pressure and sympathetic tone is also well-established (Guyenet, 2006; Dupont and Brouwers, 2010). Brain angiotensin II (Ang II), acting through AT1Rs in different parts of the brain including the PVN and the RVLM, increases mean arterial pressure (MAP) and sympathetic nerve activity. There is clear evidence that brain Ang II increases blood pressure and sympathetic activity through stimulation of AT1Rs on spinally projecting glutamatergic neurons located in the RVLM (Hu et al., 2002; Dupont and Brouwers, 2010). Similarly, microinjection of Ang II in the PVN of the hypothalamus was repeatedly reported to increase blood pressure, HR and sympathetic activity, effects also mediated through AT1R stimulation (Tagawa and Dampney, 1999; Li et al., 2006; Dupont and Brouwers, 2010). There is also a high density of Ang II containing neurons and AT1Rs in the NTS, located presynaptically on carotid sinus afferents, on interneurons (Healy et al., 1989), and on catecholamine containing neurons (Yang et al., 1997). Microinjections of Ang II within the NTS were reported to result in decreases or increases in blood pressure depending on the volume, concentration or region of Ang II injection (Casto and Phillips, 1984a; Rettig et al., 1986; Tan et al., 2007; Zhang et al., 2009). However, current evidence provided by several research groups clearly indicates that brain Ang II modulates the central integration of baroreceptor inputs within the NTS resulting in attenuation of baroreceptor sensitivity and dampening of baroreceptor reflexes and, hence, elevated blood pressure (Matsumura et al., 1998; Ferguson et al., 2001; Wong et al., 2002; Tan et al., 2005; Yao et al., 2008).

It has been postulated that the Ang II mediated reduction in baroreceptor feedback could be due to a biasing of the transmission between baroreceptor afferents and second-order neurons in the NTS, mediated by increased inhibitory GABAergic neurotransmission (Paton et al., 2001a; Guyenet, 2006). The brain GABAergic system is indeed also known to play a key role in cardiovascular regulation (Yao et al., 2008). The NTS is densely populated with GABAergic neurons, GABAergic nerve terminals (Bailey et al., 2008) as well as with GABA_A_- and GABA_B_- receptors (Zhang et al., 2009), suggesting an important modulatory role of GABA within the NTS (Zhang et al., 2009). GABAergic neurons have been identified as the predominant intrinsic network co-ordinating cardiovascular functions within the NTS (Potts et al., 2003; Bailey et al., 2008; Dufour et al., 2010). Microinjections of GABA_B_ receptor agonists within the NTS produce a pressor response, whereas GABA_B_ receptor antagonists produce a depressor response (Vitela and Mifflin, 2001; Mei et al., 2003; Zhang et al., 2009). Zhang et al. (2009) reported that chronic intracerebroventricular (icv) infusion of Ang II increased the expression of GABA_B_ receptors within the NTS and enhanced the pressor response to a GABA_B_ receptor agonist injected into the NTS (Zhang et al., 2009). Paton et al. (2001a) reported that the attenuating effect of Ang II applied in the NTS on the baroreceptor reflex was prevented by GABA_A_ receptor antagonism with bicuculline (Paton et al., 2001a), suggesting that the dampening effect of Ang II within the NTS could be GABA dependent and that GABA_A_ receptor stimulation is involved. Hence, there is evidence indicating that actions of GABA and Ang II within the NTS on blood pressure are similar (Yao et al., 2008), suggesting that these neuromediators may interact within the NTS to influence the sympathetic tone. Nevertheless, in none of these above mentioned studies GABA concentrations were measured.

Our main objective was therefore to further test this hypothesis using microdialysis to investigate the responses evoked by Ang II infusion within the NTS in normotensive Wistar rats. We assessed blood pressure and HR changes and possible effects on local glutamate and GABA concentrations in response to unilateral administration of Ang II within the NTS. Further, as Ang II and GABA are both known to interact with nitric oxide (NO) in other brain area’s (Dupont and Brouwers, 2010), and as NO was suggested to be involved in the Ang II mediated modulation of the baroreceptor reflex pathway (Paton et al., 2001a), we also aimed to investigate the possible role of the NO-pathway in the responses evoked by AT1R activation within the NTS.

## Materials and Methods

### Animals

All experiments were performed on normotensive male albino Wistar rats (Charles River Laboratories, L’Arbresle, France) weighing 250–300 g at time of surgery. Animals acclimatized at least 1 week to the animal facility before surgery at constant temperature (22 ± 3°C), a relative humidity of 55% ( ± 10%), a 12 h light–dark cycle and with free access to water and food (Safe A_04_ maintenance diet). The sodium content of the food was 2.500 mg/kg implying that the daily sodium intake was approximately 54 mg per day. All procedures used and described for animal experiments were carried out in accordance with the National and European guidelines for animal experimental research (2010/63/EU) and were approved by the Ethical Committee for Animal Experiments of the Vrije Universiteit Brussel, Belgium (Project No. 18-213-1). All efforts were made to avoid animal suffering. To minimize the number of animals used, each animal was used for microdialysis sampling as well as for MAP measurement and a control/baseline period before – and a wash out period after compound administration in the same animal. The number of rats used for each experiment was based on power calculations, which were approved by the Ethical Committee. In accordance with the ARRIVE Guidelines (Kilkenny et al., 2010) and the general policy at our institution, the number of animals used for each experiment was kept to the minimum required to allow for clear and valid conclusions.

### Drugs

Ang II, bicuculline and *N*(ω)-nitro-L-arginine methyl ester (L-NAME) were purchased from Sigma Aldrich, Co. (St. Louis, MO, United States). Ang II was used as an AT1R agonist, bicuculline as a GABA_A_ receptor antagonist and L-NAME as an inhibitor of NO-synthase (NOS). Candesartan was used as a competitive-insurmountable AT1R antagonist (Tocris Bioscience, Bristol, United Kingdom). Infusion doses of Ang II, bicuculline, L-NAME and candesartan were selected based on previous studies (Ogawa et al., 1995; Smolders et al., 1995a; Mertens et al., 2010; Brouwers et al., 2015).

The treatment compounds [Ang II (1 and 3 μg μL^−1^ h^−1^), candesartan (0.5 and 1.5 ng μL^−1^h^−1^), Ang II (1 and 3 μg μL^−1^ h^−1^) + candesartan (0.5 ng μL^−1^ h^−1^), Ang II (3 μg μL^−1^ h^−1^) + candesartan (1.5 ng μL^−1^ h^−1^), Ang II (3 μg μL^−1^h^−1^) + bicuculline (4 μg μL^−1^ h^−1^), bicuculline (4 μg μL^−1^ h^−1^), Ang II (3 μg μL^−1^ h^−1^) + L-NAME (0.4 μg μL^−1^ h^−1^), L-NAME (0.4 μg μL^−1^ h^−1^)] were dissolved in modified Ringer’s solution (147 mM NaCl, 2.3 mM CaCl_2_, 4 mM KCl) and perfused at a flow rate of 2 μL min^−1^ through the microdialysis probe.

### Experimental Design (Figure 1)

Day 1: Following acclimatization, rats were subjected to stereotactic implantation of a microdialysis guide cannula as described in section “Surgery.” A guide cannula was replaced by a microdialysis probe to locally infuse drugs and collect dialysate for the measurement of neurotransmitters. After surgery, the animals recovered overnight.

**FIGURE 1:**
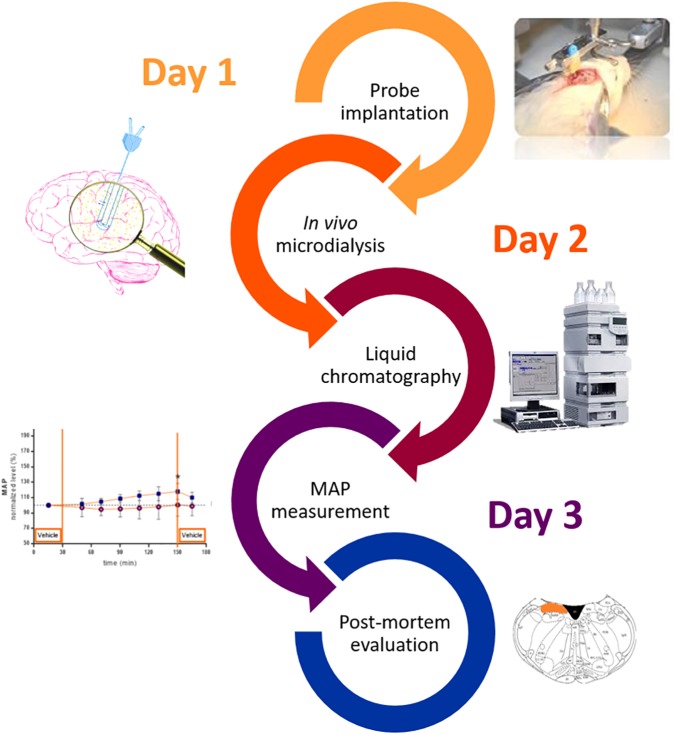
Overview experimental design.

Day 2: Microdialyis should restricted to a narrow time window (16–48 h) in order to reestablish blood brain barrier integrity and preceed gliotic reactions. Based on several studies (Benveniste and Diemer, 1987; Grabb et al., 1998; Schiffer et al., 2006; Sumbria et al., 2011) microdialysis sampling was initiated 24 h after probe implantation. All samples were analyzed by liquid chomatography (LC) for measurement of glutamate and GABA.

Day 3: The same rats as used for microdialysis experiments were anesthetized prior to cannulation of the right carotid artery for continuous monitor the MAP and HR as described in section “Mean Arterial Pressure and Heart Rate Measurement.”

### Surgery

On the first day of the experiment, all animals were anesthetized prior to surgery with ketamine/diazepam mixture (90/4.5 mg/kg) intraperitoneally and received 4 mg/kg ketoprofen subcutaneously to prevent post-operative pain and inflammation. A stainless steel microdialysis guide cannula (CMA12, Solna, Sweden) was stereotactically implanted 2 mm above the left NTS (from bregma: anterior–posterior: 1.30 mm, medial-lateral: −12.8 mm, and −5.6 mm below dura mater), as described previously (Légat et al., 2017).

### *In vivo* Microdialysis Experiment

After recovery from the stereotactical guide implantation, rats were single housed in experimental cages with free access to food and water and the microdialysis probe (CMA12/1 mm membrane length, Solna, Sweden) was inserted into the guide-cannula. The microdialysis probe was connected by tubings (Microbiotech/se AB, Stockholm, Sweden) to a micro-injection pump (BAS MD-1001). The microdialysis probe was continuously perfused overnight at a flow rate of 1 μL min^−1^ with modified Ringer’s solution (147 mM NaCl, 2.3 mM CaCl_2_, 4 mM KCl). On the second day, dialysis samples were collected from freely moving rats allowing us to measure neurotransmitters at many time points in each animal. Samples were taken every 20 min and each experiment lasted for 6 h. Each experiment started with the collection of intracerebral baseline samples (1–6) for 2 h, during which the perfusion fluid was composed of modified Ringer’s solution. Consecutively, treatment compounds dissolved in modified Ringer’s solution were perfused through the microdialysis probe from the 7^th^ collection period onward until the 12^th^ collection. Finally, samples (13–18) were taken under infusion of Ringer’s solution alone.

### Liquid Chromatography Method for Glutamate and GABA Analysis

Glutamate and GABA concentrations were measured in the dialysates using respectively, reversed-phase narrowbore LC with gradient elution and fluorescence detection and reversed-phase microbore LC with isocratic elution and electrochemical detection, as described in detail in previous publications (Smolders et al., 1995b; Van Hemelrijck et al., 2005; Légat et al., 2017).

### Mean Arterial Pressure and Heart Rate Measurement

On the third day of the experiment, animals were anesthetized by 4% sevuflurane gas in order to continuously record MAP and HR via cannulation of the right carotid artery using a pressure transducer connected to a monitor (Phillips IntelliVue MP50) as described previously (Légat et al., 2017). The right jugular vein was catheterized for fluid maintenance (0.9% NaCl). The experimental protocol started after a 30 min equilibration period following cannulation. Anesthesia was maintained by 2% sevoflurane administration. First the baseline values were recorded for 30 min before the 2 h administration of the compounds. Finally, values were recorded under infusion of Ringer’s solution alone during 30 min.

### Post-mortem Evaluation

At the end of every experiment, rats were sacrificed by an overdose of pentobarbital (Dolethal, Vétoquinol, Lure, France). Removed rat brains were kept on 4% paraformaldehyde solution. Probe localization was histologically verified after brain slicing by a neutral red staining compared against an anatomic atlas (Paxinos and Watson, 1998) in order to exclude animals with inaccurately implanted probes (Supplementary Figure S1).

### Data Analyses and Statistics

Statistical analyses were performed using GraphPad Prism 6.01 (GraphPad Software, Inc., San Diego, CA, United States) with the α level chosen at 0.05. Data are expressed as mean ± standard error of the mean (SEM). The mean values of the basal microdialysis samples as well as of the baseline MAP and HR obtained before compound administration were considered as the 100% baseline value for each animal. Results for the neurotransmitters, glutamate and GABA, were expressed as percentages of the baseline levels ± SEM. Subsequently, for the microdialysis results, an area under the curve (AUC) analysis was performed to determine if there was a difference in GABA concentrations. AUC values, expressed in arbitrary units, were compared by a Friedman test with Dunnett’s multiple comparison *post hoc* test. All MAP and HR measurements were also shown as percentage of the baseline levels ± SEM. To determine intragroup differences, data were analyzed applying a Friedman test followed by the Dunnett’s multiple comparison test.

## Results

### Angiotensin II Within the NTS Increases Extracellular GABA Levels

**FIGURE 2:**
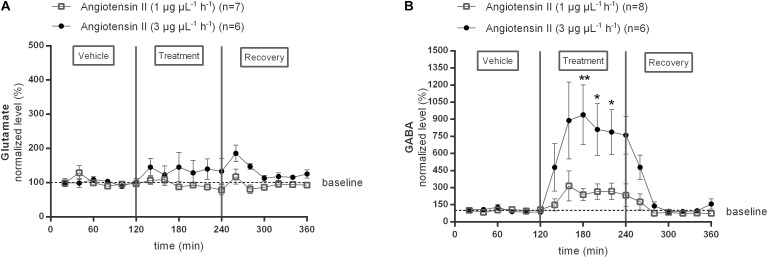
Effect of Ang II infusion (120–240 min) within the NTS on the extracellular glutamate **(A)** and GABA **(B)** concentrations in normotensive freely moving Wistar rats. Baseline values for glutamate and GABA were respectively, 402 ± 66 and 14 ± 6 nM (Ang II; 1 μg μL^−1^ h^−1^); 747 ± 55 and 16 ± 4 nM (Ang II; 3 μg μL^−1^ h^−1^). Data are presented as mean percentage of baseline levels ± SEM. Statistical analysis is performed using the Friedman test and the Dunnett’s mutiple comparison test. Local Ang II (3 μg μL^−1^ h^−1^) infusion significantly increased GABA levels, with ^∗^*p* < 0.05, ^∗∗^*p* < 0.01 compared to vehicle control.

In a first series of experiments we investigated the effect of local Ang II infusion in two different doses (1 or 3 μg μL^−1^ h^−1^) within the NTS on extracellular glutamate (Figure 2A) and GABA (Figure 2B) levels. Extracellular neurotransmitter concentrations were monitored under basal condition (0–120 min) as well as during and after administration of Ang II (120–240 min). No changes from baseline concentrations were seen for glutamate levels during Ang II infusion within the NTS [*n* = 7 (1 μg μL^−1^ h^−1^); *n* = 6 (3 μg μL^−1^ h^−1^); Figure 2A]. GABA levels tended to increase during infusion of 1 μg μL^−1^ h^−1^ Ang II within the NTS (*n* = 8; Figure 2B). Infusion of 3 μg μL^−1^ h^−1^ Ang II within the NTS induced a significant increase in GABA levels (*n* = 6; *p* < 0.05–0.01 compared to vehicle; Figure 2B).

### Angiotensin II-Evoked Increase in GABA Is Abolished by AT1R Antagonism Within the NTS

**FIGURE 3:**
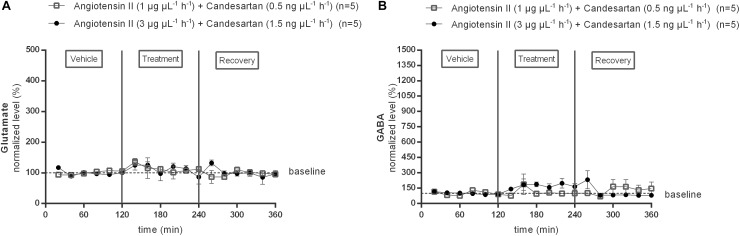
Effect of co-infusion of candesartan with Ang II (120–240 min) within the NTS on the extracellular glutamate **(A)** and GABA **(B)** concentrations in normotensive freely moving Wistar rats. Baseline values for glutamate and GABA were respectively, 724 ± 124 and 12 ± 8 nM (Ang II; 1 μg μL^−1^ h^−1^ + candesartan 0.5 ng μL^−1^ h^−1^); 473 ± 217 and 17 ± 11 nM (Ang II; 3 μg μL^−1^ h^−1^ + candesartan 1.5 ng μL^−1^ h^−1^). Data are presented as the mean percentage of baseline levels ± SEM. Statistical analysis is performed using the Friedman test and the Dunnett’s mutiple comparison test.

Subsequently, the responses to two different doses (0.5 or 1.5 ng μL^−1^ h^−1^) of the AT1R antagonist candesartan alone and during co-infusion with the two doses of Ang II were tested (Figure 3). Extracellular glutamate and GABA levels were not significantly different from the mean baseline levels during infusion with either of the two different doses (1 or 3 μg μL^−1^ h^−1^) of candesartan alone (data not shown). The previously observed trend to an increase in GABA during infusion of 1 μg μL^−1^ h^−1^ Ang II was not seen during co-infusion with 0.5 ng μL^−1^ h^−1^ candesartan (*n* = 5; Figure 3B). The GABA response to 3 μg μL^−1^ h^−1^ Ang II was attenuated and not statistically significant during co-infusion with 0.5 ng μL^−1^ h^−1^ candesartan (data not shown). Glutamate concentrations were not altered by co-infusion of 0.5 ng μL^−1^ h^−1^ candesartan with 1 μg μL^−1^ h^−1^ Ang II (*n* = 5; Figure 3A). Co-infusion of 1.5 ng μL^−1^ h^−1^ of AT1R antagonist, candesartan, with 3 μg μL^−1^ h^−1^ Ang II did not alter the glutamate concentrations (*n* = 5; Figure 3A), but abolished the Ang II evoked increase in GABA concentrations (*n* = 5; Figure 3B) within the NTS.

**FIGURE 4:**
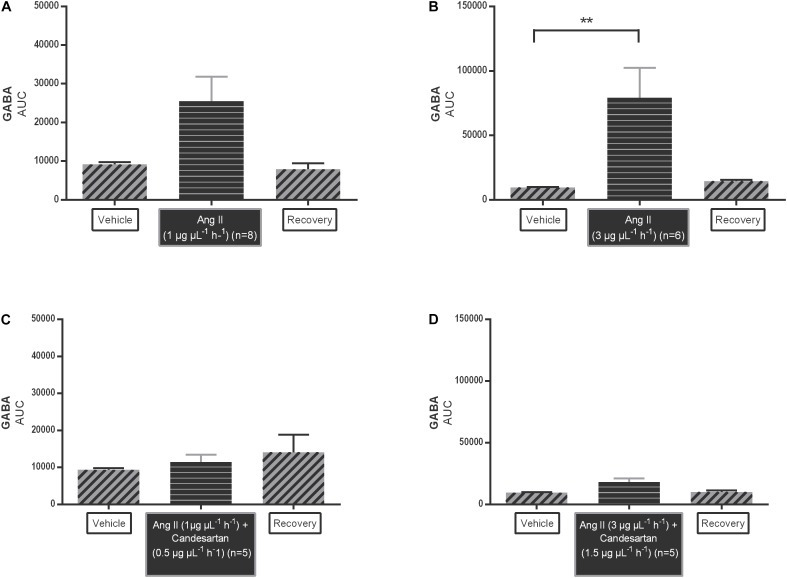
Area under the curve (AUC) values of Ang II infusion [1 μg μL^−1^ h^−1^ **(A)** and 3 μg μL^−1^ h^−1^ **(B)**] and in co-infusion with candesartan [0.5 ng μL^−1^ h^−1^ **(C)** and 1.5 ng μL^−1^ h^−1^ **(D)**] applied by microdialysis within the NTS in normotensive freely moving Wistar rats on extracellular GABA. Data are represented as the mean AUC ± SEM. Statistical analysis is performed using the Friedman test with Dunnett’s multiple comparison test. Local Ang II (3 μg μL^−1^ h^−1^) infusion significantly increased GABA levels, with ^∗∗^*p* < 0.01 compared to vehicle control.

The AUC values of GABA dialysate levels tended to increase during 1 μg μL^−1^ h^−1^ Ang II infusion (*n* = 8; Figure 4A); the higher dose of Ang II (3 μg μL^−1^ h^−1^) significantly increased the GABA levels (*n* = 6; *p* < 0.01 compared to vehicle; Figure 4B). The AUC values of GABA during co-infusion of Ang II with 0.5 ng μL^−1^ h^−1^ candesartan (*n* = 5; Figure 4C) or 1.5 ng μL^−1^ h^−1^ (*n* = 5; Figure 4D) were not different compared to the AUC values during vehicle infusion, indicating that the GABA increase induced by the higher dose of Ang II was abolished by local AT1R antagonism with high dose candesartan.

### AT1R-Evoked Increase in GABA Is Abolished by NO Synthase Inhibition Within the NTS

**FIGURE 5:**
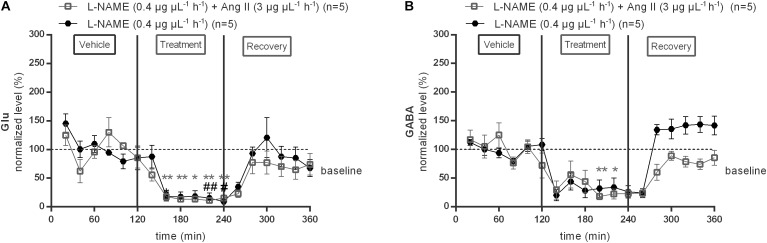
Effect of co-infusion of L-NAME with Ang II (120–240 min) within the NTS on the extracellular glutamate **(A)** and GABA **(B)** concentrations in normotensive freely moving Wistar rats. Baseline values for glutamate and GABA were respectively, 287 ± 113 and 15 ± 6 nM (L-NAME; 0.4 μg μL^−1^ h^−1^); 1431 ± 149 and 10 ± 4 nM (Ang II; 3 μg μL^−1^ h^−1^+ L-NAME 0.4 μg μL^−1^ h^−1^). Data are presented as the mean percentage of baseline levels vehicle ± SEM. Statistical analysis is performed using the Friedman test and the Dunnett’s mutiple comparison test. Local L-NAME (0.4 μg μL^−1^ h^−1^) infusion significantly decreased glutamate and GABA levels, with ^#^*p* < 0.05; ^##^*p* < 0.01 compared to vehicle control. Local L-NAME (0.4 μg μL^−1^ h^−1^) in co-infusion with Ang II (3 μg μL^−1^ h^−1^) significantly decreased glutamate levels, with ^∗^*p* < 0.05; ^∗∗^*p* < 0.01 compared to vehicle control.

To evaluate the possible involvement of NO in the AT1R mediated GABA release within the NTS, the response to the NOS inhibitor, L-NAME (0.4 μg μL^−1^ h^−1^), was tested alone and during co-infusion with 3 μg μL^−1^ h^−1^ Ang II (Figure 5). L-NAME (0.4 μg μL^−1^ h^−1^) alone significantly (*p* < 0.05–0.01 compared to vehicle) reduced baseline glutamate levels (*n* = 5; Figure 5A). Glutamate levels during co-infusion of 0.4 μg μL^−1^ h^−1^ L-NAME with 3 μg μL^−1^ h^−1^ Ang II were also significantly (*p* < 0.05–0.01 compared to vehicle) reduced but not different from levels during L-NAME (0.4 μg μL^−1^ h^−1^) alone (*n* = 5; Figure 5A). Infusion of 0.4 μg μL^−1^ h^−1^ L-NAME alone tended to decrease GABA levels (*n* = 5; Figure 5B), co-infusion of L-NAME with 3 μg μL^−1^ h^−1^ Ang II significantly (*p* < 0.05–0.01 compared to vehicle) decreased the GABA levels and reversed the Ang II evoked increase in GABA within the NTS (*n* = 5; Figure 5B).

### MAP and HR Response to Ang II Infusion Within the NTS

**FIGURE 6:**
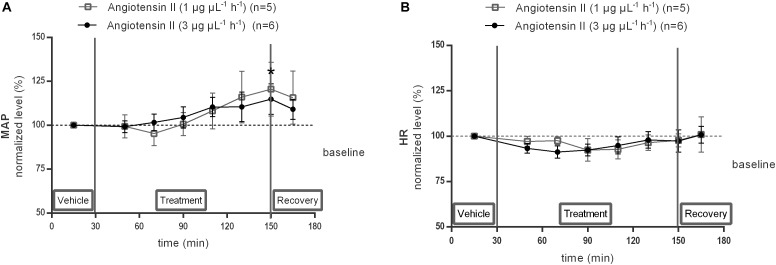
Effect of Ang II infusion (120–240 min) within the NTS on MAP **(A)** and HR **(B)** in anesthetized normotensive Wistar rats. Baseline MAP and HR were respectively, 84 ± 24 mmHg and 434 ± 19 bpm (Ang II; 1 μg μL^−1^ h^−1^); 82 ± 9 mmHg and 410 ± 83 bpm (Ang II; 3 μg μL^−1^ h^−1^). Data are presented as the mean percentage of baseline levels ± SEM. Statistical analysis is performed using the Friedman test and the Dunnett’s mutiple comparison test. Local Ang II (3 μg μL^−1^ h^−1^) infusion significantly increased MAP, with ^∗^*p* < 0.05 compared to vehicle control.

The effect of local 1 or 3 μg μL^−1^ h^−1^ Ang II infusion through microdialysis within the NTS on MAP (Figure 6A) and HR (Figure 6B) was assessed under anesthesia in the same rats, equipped with a microdialysis probe within the NTS, as used for the previous experiment. MAP tended to increase during infusion of 1 μg μL^−1^ h^−1^ Ang II within the NTS (*n* = 5; Figure 6A) and increased significantly during infusion of 3 μg μL^−1^ h^−1^ Ang II at time point 150 min (+15 mmHg compared to vehicle after 120 min Ang II infusion; *n* = 6; *p* < 0.05; Figure 6A). HR did not change during infusion of 1 μg μL^−1^ h^−1^ (*n* = 5) or 3 μg μL^−1^ h^−1^ (*n* = 6) of Ang II compared to vehicle (Figure 6B).

### MAP and HR Response to Co-infusion of Ang II With Candesartan Within the NTS

**FIGURE 7:**
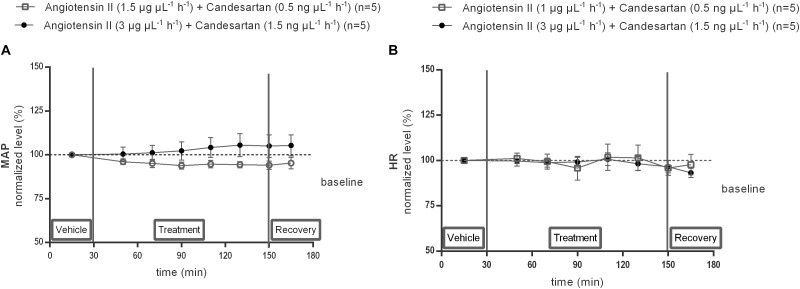
Effect of co-infusion of candesartan with Ang II (120–240 min) within the NTS on MAP **(A)** and HR **(B)** in anesthetized normotensive Wistar rats. Baseline MAP and HR were respectively, 97 ± 15 mmHg and 430 ± 46 bpm (Ang II; 1 μg μL^−1^ h^−1^ + candesartan 0.5 ng μL^−1^ h^−1^); 85 ± 12 mmHg and 412 ± 34 bpm (Ang II; 3 μg μL^−1^ h^−1^) + candesartan 1.5 ng μL^−1^ h^−1^). Data are presented as the mean percentage of baseline levels ± SEM. Statistical analysis is performed using the Friedman test and the Dunnett’s mutiple comparison test.

The responses of MAP (Figure 7A) and HR (Figure 7B) to 0.5 or 1.5 ng μL^−1^ h^−1^ of the AT1R antagonist candesartan alone and during co-infusion within the NTS with 1 or 3 μg μL^−1^ h^−1^ of Ang II were tested. Local infusion of candesartan alone within the NTS did not modify the baseline MAP nor HR (data not shown). There were no significant differences in MAP nor HR between vehicle and co-infusion of 1.5 ng μL^−1^ h^−1^ candesartan with 3 μg μL^−1^ h^−1^ Ang II (*n* = 5; Figure 7A,B), indicating that the hypertensive response to 3 μg μL^−1^ h^−1^ of Ang II alone was abolished by AT1R antagonism (Figure 7A).

### MAP and HR Response to Co-infusion of Ang II With the GABA_A_ Receptor Antagonist Bicuculline Within the NTS

**FIGURE 8:**
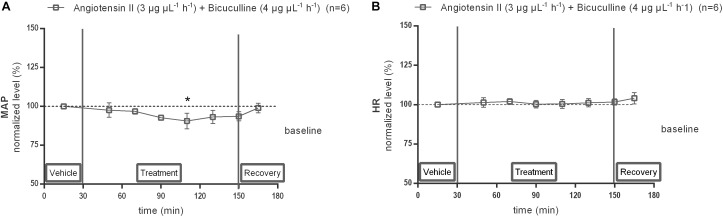
Effect of co-infusion of bicuculline with Ang II (120–240 min) within the NTS on MAP **(A)** and HR **(B)**. Baseline MAP and HR were respectively, 87 ± 11 mmHg and 411 ± 50 bpm (Ang II; 3 μg μL^−1^ h^−1^ + bicuculline 4 μg μL^−1^ h^−1^). Data are presented as the mean percentage of baseline levels vehicle ± SEM. Statistical analysis is performed using the Friedman test and the Dunnett’s mutiple comparison test. Local co-infusion of bicuculline (4 μg μL^−1^ h^−1^) with Ang II (3 μg μL^−1^ h^−1^) significantly decreased MAP, with ^∗^*p* < 0.05 compared to vehicle control.

Local infusion of GABA_A_ receptor antagonist, bicuculline (4 μg μL^−1^ h^−1^), alone within the NTS did not modify the baseline MAP nor HR (data not shown). Co-infusion of 4 μg μL^−1^ h^−1^ bicuculline with 3 μg μL^−1^ h^−1^ Ang II significantly decreased the MAP at time point 110 min (−7 mmHg compared to vehicle after 80 min co-infusion of Ang II with bicuculline; *n* = 6; *p* < 0.05; Figure 8A) and reversed the Ang II evoked increase in MAP seen in Figure 6A, but did not affect the HR (*n* = 6; Figure 8B).

### MAP and HR Response to Co-infusion of Ang II With the NO-Synthase Inhibitor L-NAME Within the NTS

**FIGURE 9:**
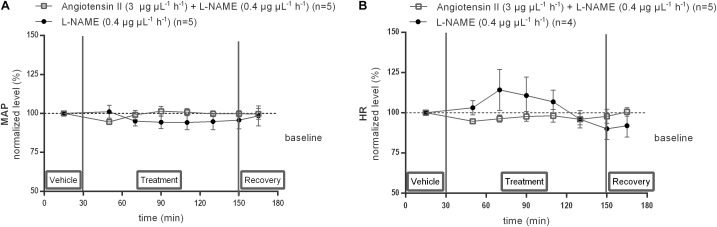
Effect of co-infusion of L-NAME with Ang II (120–240 min) within the NTS on MAP **(A)** and HR **(B)**. Baseline MAP and HR were respectively, 91 ± 10 mmHg and 477 ± 112 bpm (L-NAME 0.4 μg μL^−1^ h^−1^); 98 ± 21 mmHg and 401 ± 72 bpm (Ang II; 3 μg μL^−1^ h^−1^ + L-NAME 0.4 μg μL^−1^ h^−1^). Data are shown as the mean percentage of baseline levels ± SEM. Statistical analysis is performed using the Friedman test and the Dunnett’s mutiple comparison test.

Local infusion of L-NAME (0.4 μg μL^−1^ h^−1^) alone within the NTS did not modify baseline MAP (*n* = 5) nor HR (*n* = 4). Co-infusion of L-NAME abolished the MAP increase induced by 3 μg μL^−1^ h^−1^ Ang II (*n* = 5; Figure 9A,B).

## Discussion

The present study in male normotensive rats focused on the responses elicited by local Ang II administration within the NTS, the principal site for the termination of baroreceptor afferent fibers mediating the inhibitory effect of baroreceptors on sympathetic outflow. It is well-established from earlier studies by several groups of investigators that microinjection of Ang II into the NTS blunts the sensitivity of the baroreflex (Averill and Diz, 2000; Wong et al., 2002; Zhang et al., 2009) and that AT1R as well as Ang II containing neurons are present in the NTS (Rettig et al., 1986; Healy et al., 1989). The results of our study provide for the first time direct evidence that Ang II, acting on local AT1Rs, increases local GABA concentrations within the NTS, suggesting that the blood pressure increase which was also observed after Ang II administration is GABA dependent. This is corroborated by the observation that the blood pressure response to Ang II is abolished by co-infusion with a selective GABA_A_ receptor antagonist. GABA_A_ receptor antagonism alone did not affect blood pressure nor HR when administered unilaterally within NTS, which is in line with previous observations from Shih and Chuang (2007) reporting no changes in blood pressure and HR even after bilateral GABA_A_ receptor antagonism within the NTS (Shih and Chuang, 2007). Further, our results also show that local NO is involved in the GABA response to AT1R stimulation within the NTS.

We observed, in anesthetized rats, no change in blood pressure nor HR in response to unilateral administration of 1 μg μL^−1^ h^−1^ of Ang II, but a significant increase in blood pressure without a change in HR after infusion of 3 μg μL^−1^ h^−1^ of Ang II. These blood pressure changes were abolished by co-infusion of the selective AT1R antagonist candesartan. These results appear to be in line with earlier studies by other groups on the cardiovascular effects of microinjections of Ang II into the NTS (Rettig et al., 1986). Rettig et al. (1986) reported that microinjections of a low dose of Ang II resulted in a depressor response, whereas higher doses were characterized by a biphasic response, with an initial short lasting depressor response followed by a subsequent increase in blood pressure (Rettig et al., 1986). In our experiments, where no bolus injection of Ang II was given but where Ang II was infused through the microdialysis probe, a putative initial transitory depressor phase was probably undetectable, but we observed also a progressive increase in blood pressure. Other research groups have reported either depressor or pressor responses to microinjections of Ang II (Casto and Phillips, 1984a,b). Overall, it appears from different studies with microinjections of Ang II into the NTS that responses to Ang II display a complex dose-response relationship and that it can produce decreases or increases in blood pressure depending on the infusion dose used, the volume injected, and the exact region of the injection (Yao et al., 2008; Zhang et al., 2009). Such qualitatively different responses could be conceivable with the hypothesis that Ang II may not act as a neurotransmitter itself but rather as a neuromodulator within the NTS, and that effects may differ according to differences in activation states of the different neurons and pathways involved in the regulation of sympathetic tone.

Our observation that central administration of Ang II increased blood pressure without concomitant elevation of HR was also previously reported by several other investigators, even after bilateral injection (Bruner et al., 1985; Sagara et al., 2007; Zhang et al., 2009). A possible explanation is that the Ang II mediated increase in blood pressure may trigger baroreflex mediated cardiovascular responses, buffering a putative tachycardic response and also dampening the blood pressure increase, in particular as the contralateral NTS remained intact during our experiments with unilateral administration of Ang II.

The major novel finding of the present study is that local administration of Ang II using microdialysis in conscious normotensive rats induced a significant increase in local GABA concentrations within the NTS. The GABA concentrations returned to baseline levels shortly after stopping the Ang II infusion. The GABA response to Ang II was completely abolished by local co-infusion with the AT1R antagonist candesartan, indicating that this GABA response was mediated through stimulation of local AT1R. Whereas candesartan abolished the responses evoked by exogenous Ang II, we did not observe an effect of candesartan itself on blood pressure or GABA levels. This suggests that, at least in our experimental setting, endogenous activation of AT1R within the NTS does not appear to contribute significantly to the control of GABA release and blood pressure in these baseline conditions in normotensive rats. Ang II and the AT1R antagonist did not significantly affect local glutamate concentrations in the NTS.

It is therefore tempting to postulate that the well-established dampening effect on the baroreceptor reflex of local administration of Ang II is dependent on this AT1R mediated increased release of GABA. Reduction in baroreceptor feedback could indeed be due to inhibition of the transmission between baroreceptor afferents and second-order neurons in the NTS, mediated by pre-and post-synaptic inhibition by GABA (Guyenet, 2006). GABAergic systems are indeed known to play a key role in cardiovascular regulation via GABA_A_ and GABA_B_ receptors in the NTS (Yao et al., 2008). GABA was shown to be a potent modulator of neurons in the NTS, which contains a high density of GABA_A_ and GABA_B_ receptor containing neurons and a high density of GABA containing nerve terminals (Zhang et al., 2009). GABA receptor agonists elicit a pressor response when injected into the NTS and GABA_B_ receptor antagonism results is a depressor response (Zhang et al., 2009). Paton et al. (2001a) reported that the attenuation of the cardiac vagal and sympathetic components of the baroreceptor reflex by Ang II was antagonized by GABA_A_ receptor antagonism with bicuculline (Paton et al., 2001a). The same group of investigators also reported in an earlier paper that GABAergic synaptic transmission was potentiated by bath application of Ang II in a subpopulation of NTS neurons recorded intracellularly *in vitro* (Paton and Kasparov, 1999). Based on these *in vitro* findings, they suggested that Ang II might cause an increase in release of GABA from terminals of GABAergic neurons (Paton and Kasparov, 1999). Our *in vivo* results are in line with this hypothesis.

Taken together, these findings reported in the literature combined with our novel direct observation of AT1R mediated increase in GABA concentrations within the NTS suggest that GABA has a crucial role in the dampening effect of Ang II on the baroreceptor reflex.

In normal physiological circumstances, in the absence of exogenous Ang II administration, an increase in blood pressure activates baroreceptors located in the vessel wall of the arteria carotis and the aortic arch, ultimately leading to suppression of the sympathetic tone. The baroreceptor afferents activate NTS efferent glutamatergic neurons which connect to and activate the GABAergic neurons within the CVLM. These GABAergic nerve terminals originating in the CVLM inhibit the activity of the glutamatergic neurons within the RVLM that drive the sympathetic tone. Hence, the NTS neurons inhibit the RVLM indirectly through the activation of these inhibitory GABAergic nerve terminals originating in the CVLM (Guyenet, 2006). Local GABA release within the NTS, as induced by AT1R stimulation by Ang II, can be expected to increase inhibitory GABAergic neurotransmission and reduce the activity of glutamatergic NTS neurons and, subsequently, the activation of the inhibitory GABA neurons in the CVLM, leading to less suppression of the RVLM neurons that drive the sympathetic tone. The final result is inhibition of baroreceptor afferent input signals triggered by increased blood pressure and less suppression of sympathetic tone or, in other words, the well-documented dampening of the baroreflex by Ang II. Through this GABA dependent mechanism, with increased release of GABA within the NTS and, indirectly, reduced release from GABA by CVLM neurons, Ang II can interfere with short term blood pressure fluctuations, but could also lead to a baroreceptor resetting within the NTS resulting in an upregulation of the activity of the RVLM neurons and development of hypertension.

Based on the current evidence, we could hypothesize that Ang II directly activates presynaptic AT1R located on GABAergic (inter)neurons within the NTS resulting in an increase in GABA release. An alternative possibility is that the Ang II mediated release of GABA from local inhibitory GABAergic interneurons within the NTS occurs indirectly and that Ang II stimulates the release of NO which is then responsible for increased GABA release. Our observation that the AT1R mediated increase in GABA within the NTS is abolished by co-infusion of the NOS inhibitor L-NAME supports this latter possibility and is also in line with previous evidence from different research groups (Paton et al., 2001a; Guyenet, 2006).

Ang II can indeed stimulate NO release (Millatt et al., 1999), and an NO donor microinjected into the NTS was shown to depress the baroreceptor reflex in an *in situ* ‘working heart-brainstem preparation’ (Paton et al., 2001a). Moreover, microinjection of *N*(ω)-methyl-L-arginine acetate (L-NMMA), another NOS inhibitor, was shown by Paton et al. (2001a) to abolish the effect of Ang II on baroreflex function (Paton et al., 2001a). Our observation that NOS inhibition abolished the GABA response and the blood pressure response to Ang II administered into the NTS indicates that this AT1R mediated effect within the NTS is dependent on local NO release, either from neuronal (n) or endothelial (e) origin. NO producing cells and both, n-NOS and e-NOS, are indeed present within the NTS, and AT1Rs were reported to be co-localized with e-NOS in both neurons and endothelial cells (Dun et al., 1994, 1995; Paton et al., 2001a). Previous studies suggest that also circulating Ang II may modulate the baroreflex (Tan et al., 2007; Paton et al., 2008). Circulating Ang II was indeed suggested to reduce the transmission between baroreceptor afferents and NTS efferent neurons by activation of endothelial AT1R, causing the release of NO which in turn potentiates GABA release within the NTS (Paton et al., 2001a,b; Guyenet, 2006). Similarly, NO has also been shown to inhibit neuronal activity through enhanced GABA release in other centers within the medulla oblongata including the PVN (Li et al., 2002, 2003, 2004).

We also observed that local administration of the NOS inhibitor L-NAME alone tended to decrease GABA concentrations within the NTS suggesting the presence of an endogenous tone stimulating GABA release, in agreement with earlier suggestions based on gene transfer studies in conscious rats that eNOS is constitutively active within the NTS in conscious rats as a factor regulating baroreceptor reflex gain (Waki et al., 2003). This is also in line with earlier observations of Li et al. (2003), using *in vitro* patch clamp recordings from RVLM projecting PVN neurons, that basal GABAergic neuronal firing activity was reduced by NOS inhibition (Li et al., 2003). It is also consistent with previous *in vivo* studies showing that NOS inhibitors within the PVN increase renal sympathetic discharge (Zhang et al., 1997, 2001).

Our observation that NOS inhibition *per se* also significantly reduced baseline glutamate levels within the NTS suggests that endogenously formed NO also stimulates glutamate release, and is in line with earlier findings by Matsuo et al. (2001) that NO production, evoked by *N*-methyl-D aspartate (NMDA) receptor stimulation, can increase glutamate release within the NTS (Matsuo et al., 2001). In the hippocampus also, NO formation in response to NMDA receptor activation was reported to enhance presynaptic glutamate release trough increased cyclic guanosine monophosphate formation (Neitz et al., 2011). Our results therefore corroborate earlier observations that NO can increase the release of GABA as well as of glutamate in different autonomic centers and, hence, is an important neuromodulator (Zanzinger, 2002).

The present study has some limitations. Sevoflurane, which was used for this anesthesia, is known to reduce blood pressure (Tsikas et al., 2015). However, our experimental protocol for MAP measurement was designed in a way to include a recovery period after cannulation of the carotid artery as well as a baseline period before test compound administration allowing us to have stable blood pressure before starting the administration of pharmacological compounds. Furthermore, the influence of sevoflurane was present and remained constant, during the whole study period: baseline, during test compound (in the control situation) and after withdrawal of test compounds. These experimental conditions allowed us to detect a hypertensive response (change from baseline) mediated by AT1R stimulation within the NTS compared to baseline measurement. Similarly, in other studies in the same experimental setting, a hypotensive response induced by clonidine administration was reproducibly demonstrated, as expected (data not shown). Therefore, even under anesthesia, short term changes in blood pressure induced by interventions such as Ang II administration, can be reproducibly detected and assessed with direct continuous monitoring of blood pressure. We used L-NAME, a non-selective NOS inhibitor in the present study to demonstrate that the AT1R mediated effect on GABA release is dependent on an intact NO pathway. It would be of interest to further investigate the NO involved is from neuronal or endothelial origin using more selective e- versus n-NOS inhibitors. In addition, as the NTS densely expresses AT2R which are mainly located on GABA neurons (de Kloet et al., 2016), further research exploring the possible effects of AT2R stimulation within the NTS on GABA levels and blood pressure seems warranted. It has indeed been hypothesized that AT2R stimulation on GABA neurons within the NTS may act to inhibit GABA release, thereby reducing the tonic inhibition of baroreflex pathways provided by these GABAergic neurons, eventually reducing blood pressure (Steckelings et al., 2017).

We conclude that Ang II mediated stimulation of AT1Rs within the NTS results in activation of either eNOS or nNOS (or both), with a subsequent local production of NO, which enhances GABA release probably from NTS interneurons. We speculate that this release of GABA within the NTS inhibits glutamatergic neurons projecting to the CVLM, thereby reducing the activation of the CVLM inhibitory GABAergic nerves with subsequent disinhibition of the sympathetic driving glutamatergic neurons in the RVLM. Our results further suggest that NO produced within the NTS tonically potentiates local GABA and glutamate release, and may therefore also be an important modulator of the central blood pressure regulation.

## Ethics Statement

This study was carried out in accordance with the recommendations of “National and European guidelines for animal experimental research, Ethical Committee for Animal Experiments of the Vrije Universiteit Brussel, Belgium.” The protocol was approved by the “Ethical Committee for Animal Experiments of the Vrije Universiteit Brussel, Belgium.”

## Author Contributions

LL performed the research and wrote the research manuscript. IS and AD designed the research study and gave critical insights in the research manuscript.

## Conflict of Interest Statement

The authors declare that the research was conducted in the absence of any commercial or financial relationships that could be construed as a potential conflict of interest.

## Supplementary Material

FIGURE S1 Histological verification of the probe localization by a neutral red staining compared against an anatomic atlas (Paxinos and Watson, 1998).

## References

[B1] Averill D. B. Diz D. I. (2000). Angiotensin peptides and baroreflex control of sympathetic outflow: pathways and mechanisms of the medulla oblongata. *Brain Res. Bull.* 51 119–128. 10.1016/s0361-9230(99)00237-3 10709957

[B2] Bailey T. W. Appleyard S. M. Jin Y. H. Andersen M. C. (2008). Organization and properties of GABAergic neurons in solitary tract nucleus (NTS). *J. Neurophysiol.* 99 1712–1722. 10.1152/jn.00038.2008 18272881

[B3] Benveniste H. Diemer N. H. (1987). Cellular reactions to implantation of a microdialysis tube in the rat hippocampus. *Acta Neuropathol.* 74 234–238. 10.1007/bf00688186 3673515

[B4] Brouwers S. Smolders I. Wainford R. D. Dupont A. G. (2015). Hypotensive and sympathoinhibitory responses to selective central AT2 receptor stimulation in spontaneously hypertensive rats. *Clin. Sci.* 129 81–92. 10.1042/CS20140776 25655919 PMC4430196

[B5] Bruner C. A. Weaver J. M. Fink G. D. (1985). Sodium-dependent hypertension produced by chronic central angiotensin II infusion. *Am. J. Physiol.* 249 H321–H327. 10.1152/ajpheart.1985.249.2.H321 4025567

[B6] Casto R. Phillips M. I. (1984a). Cardiovascular actions of microinjections of angiotensin II in the brain stem of rats. *Am. J. Physiol.* 246 R811–R816. 10.1152/ajpregu.1984.246.5.R811 6721000

[B7] Casto R. Phillips M. I. (1984b). Mechanism of pressor effects by angiotensin in the nucleus tractus solitarius of rats. *Am. J. Physiol.* 247 R575–R581. 10.1152/ajpregu.1984.247.3.R575 6089597

[B8] de Kloet A. D. Wang L. Ludin J. A. Smith J. A. Pioquinto D. J. Hiller H. (2016). Reporter mouse strain provides a novel look at angiotensin type-2 receptor distribution in the central nervous system. *Brain Struct. Funct.* 221 891–912. 10.1007/s00429-014-0943-1 25427952 PMC4446257

[B9] Dufour A. Tell F. Baude A. (2010). Perinatal development of inhibitory synapses in the nucleus tractus solitarii of the rat. *Eur. J. Neurosci.* 32 538–549. 10.1111/j.1460-9568.2010.07309.x 20718854

[B10] Dun N. J. Dun S. L. Forstermann U. (1994). Nitric oxide synthase immunoreactivity in rat pontine medullary neurons. *Neuroscience* 59 429–445. 10.1016/0306-4522(94)90607-6 7516501

[B11] Dun N. J. Dun S. L. Hwang L. L. Fôrstermann U. (1995). Infrequent co-existence of nitric oxide synthase and parvalbumin, calbindin and calretinin immunoreactivity in rat pontine neurons. *Neurosci. Lett.* 191 165–168. 10.1016/0304-3940(95)11582-h 7543992

[B12] Dupont A. G. Brouwers S. (2010). Brain angiotensin peptides regulate sympathetic tone and blood pressure. *J. Hypertens.* 28 1599–1610. 10.1097/HJH.0b013e32833af3b2 20502352

[B13] Ferguson A. V. Washburn D. L. Latchford K. J. (2001). Hormonal and neurotransmitter roles for angiotensin in the regulation of central autonomic function. *Exp. Biol. Med.* 226 85–96. 10.1177/153537020122600205 11446443

[B14] Grabb M. C. Sciotti V. M. Gidday J. M. Cohen S. A. van Wylen D. G. (1998). Neurochemical and morphological responses to acutely and chronically implanted brain microdialysis probes. *J. Neurosci. Methods* 82 25–34. 10.1016/s0165-0270(98)00025-9 10223512

[B15] Guyenet P. G. (2006). The sympathetic control of blood pressure. *Nat. Rev. Neurosci.* 7 335–346. 10.1038/nrn1902 16760914

[B16] Healy D. P. Rettig R. Nguyen T. Printz M. P. (1989). Quantitative autoradiography of angiotensin II receptors in the rat solitary-vagal area: effects of nodose ganglionectomy or sinoaortic denervation. *Brain Res.* 484 1–12. 10.1016/0006-8993(89)90343-0 2713673

[B17] Hu L. Zhu D. N. Yu Z. Wang J. Q. Sun Z. J. Yao T. (2002). Expression of angiotensin II type 1 (AT(1)) receptor in the rostral ventrolateral medulla in rats. *J. Appl. Physiol.* 92 2153–2161. 10.1152/japplphysiol.00261.2001 11960969

[B18] Kilkenny C. Browne W. J. Cuthill I. C. Emerson M. Altman D. G. (2010). Improving bioscience research reporting: the ARRIVE guidelines for reporting animal research. *PLoS Biol.* 8:e1000412. 10.1371/journal.pbio.1000412. 20613859 PMC2893951

[B19] Légat L. Brouwers S. Smolders I. Dupont A. G. (2017). Hypotensive response to angiotensin II type 2 receptor stimulation in the rostral ventrolateral medulla requires functional GABA-A receptors. *Front. Neurosci.* 11:346. 10.3389/fnins.2017.00346 28674483 PMC5474467

[B20] Li D. P. Chen S. R. Finnegan T. F. Pan H. L. (2004). Signalling pathway of nitric oxide in synaptic GABA release in the rat paraventricular nucleus. *J. Physiol.* 554 100–110. 10.1113/jphysiol.2003.053371 14678495 PMC1664752

[B21] Li D. P. Chen S. R. Pan H. L. (2002). Nitric oxide inhibits spinally projecting paraventricular neurons through potentiation of presynaptic GABA release. *J. Neurophysiol.* 88 2664–2674. 10.1152/jn.00540.2002 12424302

[B22] Li Y. Zhang W. Stern J. E. (2003). Nitric oxide inhibits the firing activity of hypothalamic paraventricular neurons that innervate the medulla oblongata: role of GABA. *Neuroscience* 118 585–601. 10.1016/s0306-4522(03)00042-3 12710969

[B23] Li Y. F. Wang W. Mayhan W. G. Patel K. P. (2006). Angiotensin-mediated increase in renal sympathetic nerve discharge within the PVN: role of nitric oxide. *Am. J. Physiol. Regul. Integr. Comp. Physiol.* 290 R1035–R1043. 10.1152/ajpregu.00338.2004 16322353

[B24] Matsumura K. Averill D. B. Ferrario C. M. (1998). Angiotensin II acts at AT1 receptors in the nucleus of the solitary tract to attenuate the baroreceptor reflex. *Am. J. Physiol.* 275 R1611–R1619. 10.1152/ajpregu.1998.275.5.R1611 9791081

[B25] Matsuo I. Hirooka Y. Hironaga K. Eshima K. Shigematsu H. Shihara M. (2001). Glutamate release via NO production evoked by NMDA in the NTS enhances hypotension and bradycardia in vivo. *Am. J. Physiol. Regul. Integr. Comp. Physiol.* 280 R1285–R1291. 10.1152/ajpregu.2001.280.5.R1285 11294745

[B26] Mei L. Zhang J. Mifflin S. (2003). Hypertension alters GABA receptor-mediated inhibition of neurons in the nucleus of the solitary tract. *Am. J. Physiol. Regul. Integr. Comp. Physiol.* 285 R1276–R1286. 10.1152/ajpregu.00255.2003 14615399

[B27] Mertens B. Vanderheyden P. Michotte Y. Sarre S. (2010). Direct angiotensin II type 2 receptor stimulation decreases dopamine synthesis in the rat striatum. *Neuropharmacology* 58 1038–1044. 10.1016/j.neuropharm.2010.01.009 20097214

[B28] Millatt L. J. Abdel-Rahman E. M. Siragy H. M. (1999). Angiotensin II and nitric oxide: a question of balance. *Regul. Pept.* 81 1–10. 10.1016/s0167-0115(99)00027-0 10395403

[B29] Neitz A. Mergia E. Eysel U. T. Koesling D. Mittmann T. (2011). Presynaptic nitric oxide/cGMP facilitates glutamate release via hyperpolarization-activated cyclic nucleotide-gated channels in the hippocampus. *Eur. J. Neurosci.* 33 1611–1621. 10.1111/j.1460-9568.2011.07654.x 21410795

[B30] Ogawa H. Mizusawa A. Kikuchi Y. Hida W. Miki H. Shirato K. (1995). Nitric oxide as a retrograde messenger in the nucleus tractus solitarii of rats during hypoxia. *J. Physiol.* 486(Pt 2), 495–504. 10.1113/jphysiol.1995.sp020828 7473213 PMC1156537

[B31] Paton J. F. Boscan P. Murphy D. Kasparov S. (2001a). Unravelling mechanisms of action of angiotensin II on cardiorespiratory function using in vivo gene transfer. *Acta Physiol. Scand.* 173 127–137. 10.1046/j.1365-201X.2001.00898.x 11678735

[B32] Paton J. F. Deuchars J. Ahmad Z. Wong L. F. Murphy D. Kasparov S. (2001b). Adenoviral vector demonstrates that angiotensin II-induced depression of the cardiac baroreflex is mediated by endothelial nitric oxide synthase in the nucleus tractus solitarii of the rat. *J. Physiol.* 531 445–458. 10.1111/j.1469-7793.2001.0445i.x 11230517 PMC2278463

[B33] Paton J. F. Kasparov S. (1999). Differential effects of angiotensin II on cardiorespiratory reflexes mediated by nucleus tractus solitarii - a microinjection study in the rat. *J. Physiol.* 521(Pt 1), 213–225. 10.1111/j.1469-7793.1999.00213.x 10562346 PMC2269655

[B34] Paton J. F. Wang S. Polson J. W. Kasparov S. (2008). Signalling across the blood brain barrier by angiotensin II: novel implications for neurogenic hypertension. *J. Mol. Med.* 86 705–710. 10.1007/s00109-008-0324-324 18443753

[B35] Paxinos G. Watson S. (1998). *The Rat Brain in Stereotaxic Coordinates.* San Diego, CA: Academic Press.

[B36] Potts J. T. Paton J. F. Mitchell J. H. Garry M. G. Kline G. Anguelov P. T. (2003). Contraction-sensitive skeletal muscle afferents inhibit arterial baroreceptor signalling in the nucleus of the solitary tract: role of intrinsic GABA interneurons. *Neuroscience* 119 201–214. 10.1016/s0306-4522(02)00953-3 12763081

[B37] Rettig R. Healy D. P. Printz M. P. (1986). Cardiovascular effects of microinjections of angiotensin II into the nucleus tractus solitarii. *Brain Res.* 364 233–240. 10.1016/0006-8993(86)90835-8 3947969

[B38] Sagara Y. Hirooka Y. Nozoe M. Ito K. Kimura Y. Sunagawa K. (2007). Pressor response induced by central angiotensin II is mediated by activation of Rho/Rho-kinase pathway via AT1 receptors. *J. Hypertens.* 25 399–406. 10.1097/HJH.0b013e328010b87f 17211247

[B39] Schiffer W. K. Mirrione M. M. Biegon A. Alexoff D. L. Patel V. Dewey S. L. (2006). Serial microPET measures of the metabolic reaction to a microdialysis probe implant. *J. Neurosci. Methods* 155 272–284. 10.1016/j.jneumeth.2006.01.027 16519945

[B40] Shih C. D. Chuang Y. C. (2007). Nitric oxide and GABA mediate bi-directional cardiovascular effects of orexin in the nucleus tractus solitarii of rats. *Neuroscience* 149 625–635. 10.1016/j.neuroscience.2007.07.016 17916408

[B41] Smolders I. De Klippel N. Sarre S. Ebinger G. Michotte Y. (1995a). Tonic GABA-ergic modulation of striatal dopamine release studied by in vivo microdialysis in the freely moving rat. *Eur. J. Pharmacol.* 284 83–91. 10.1016/0014-2999(95)00369-v 8549640

[B42] Smolders I. Sarre S. Michotte Y. Ebinger G. (1995b). The analysis of excitatory, inhibitory and other amino acids in rat brain microdialysates using microbore liquid chromatography. *J. Neurosci. Methods* 57 47–53. 10.1016/0165-0270(94)00124-y 7791364

[B43] Steckelings U. M. Kloet A. Sumners C. (2017). Centrally mediated cardiovascular actions of the angiotensin II type 2 receptor. *Trends Endocrinol. Metab.* 28 684–693. 10.1016/j.tem.2017.06.002 28733135 PMC5563271

[B44] Sumbria R. K. Klein J. Bickel U. (2011). Acute depression of energy metabolism after microdialysis probe implantation is distinct from ischemia-induced changes in mouse brain. *Neurochem. Res.* 36 109–116. 10.1007/s11064-010-0276-272 20878232

[B45] Tagawa T. Dampney R. A. (1999). AT(1) receptors mediate excitatory inputs to rostral ventrolateral medulla pressor neurons from hypothalamus. *Hypertension* 34 1301–1307. 10.1161/01.hyp.34.6.1301 10601134

[B46] Tan P. S. Killinger S. Horiuchi J. Dampney R. A. (2007). Baroreceptor reflex modulation by circulating angiotensin II is mediated by AT1 receptors in the nucleus tractus solitarius. *Am. J. Physiol. Regul. Integr. Comp. Physiol.* 293 R2267–R2278. 10.1152/ajpregu.00267.2007 17855497

[B47] Tan P. S. Potas J. R. Killinger S. Horiuchi J. Goodchild A. K. Pilowsky P. M. (2005). Angiotensin II evokes hypotension and renal sympathoinhibition from a highly restricted region in the nucleus tractus solitarii. *Brain Res.* 1036 70–76. 10.1016/j.brainres.2004.12.018 15725403

[B48] Tsikas D. Jordan J. Engeli S. (2015). Blood pressure-lowering effects of propofol or sevoflurane anaesthesia are not due to enhanced nitric oxide formation or bioavailability. *Br. J. Clin. Pharmacol.* 79 1030–1033. 10.1111/bcp.12568 25475891 PMC4456136

[B49] Van Hemelrijck A. Sarre S. Smolders S. Michotte Y. (2005). Determination of amino acids associated with cerebral ischaemia in rat brain microdialysates using narrowbore liquid chromatography and fluorescence detection. *J. Neurosci. Methods* 144 63–71. 10.1016/j.jneumeth.2004.10.013 15848240

[B50] Vitela M. Mifflin S. W. (2001). Gamma-aminobutyric acid(B) receptor-mediated responses in the nucleus tractus solitarius are altered in acute and chronic hypertension. *Hypertension* 37 619–622. 10.1161/01.hyp.37.2.619 11230345

[B51] Waki H. Kasparov S. Wong L. F. Murphy D. Shimizu T. Paton J. F. (2003). Chronic inhibition of endothelial nitric oxide synthase activity in nucleus tractus solitarii enhances baroreceptor reflex in conscious rats. *J. Physiol.* 546 233–242. 10.1113/jphysiol.2002.030270 12509491 PMC2342461

[B52] Wong L. F. Polson J. W. Murphy D. Paton J. F. Kasparov S. (2002). Genetic and pharmacological dissection of pathways involved in the angiotensin II-mediated depression of baroreflex function. *FASEB J.* 16 1595–1601. 10.1096/fj.02-0099com 12374782

[B53] Yang S. N. Lippoldt A. Jansson A. Phillips M. I. Ganten D. Fuxe K. (1997). Localization of angiotensin II AT1 receptor-like immunoreactivity in catecholaminergic neurons of the rat medulla oblongata. *Neuroscience* 81 503–515. 10.1016/s0306-4522(97)00057-2 9300437

[B54] Yao F. Sumners C. O’Rourke S. T. Sun C. (2008). Angiotensin II increases GABAB receptor expression in nucleus tractus solitarii of rats. *Am. J. Physiol. Heart Circ. Physiol.* 294 H2712–H2720. 10.1152/ajpheart.00729.2007 18424635 PMC4422374

[B55] Zanzinger J. (2002). Mechanisms of action of nitric oxide in the brain stem: role of oxidative stress. *Auton. Neurosci.* 98 24–27. 10.1016/s1566-0702(02)00025-5 12144034

[B56] Zhang K. Li Y. F. Patel K. P. (2001). Blunted nitric oxide-mediated inhibition of renal nerve discharge within PVN of rats with heart failure. *Am. J. Physiol. Heart Circ. Physiol.* 281 H995–H1004. 10.1152/ajpheart.2001.281.3.H995 11514264

[B57] Zhang K. Mayhan W. G. Patel K. P. (1997). Nitric oxide within the paraventricular nucleus mediates changes in renal sympathetic nerve activity. *Am. J. Physiol.* 273 R864–R872. 10.1152/ajpregu.1997.273.3.R864 9321861

[B58] Zhang Q. Yao F. O’Rourke S. T. Qian S. Y. Sun C. (2009). Angiotensin II enhances GABA(B) receptor-mediated responses and expression in nucleus tractus solitarii of rats. *Am. J. Physiol. Heart Circ. Physiol.* 297 H1837–H1844. 10.1152/ajpheart.00354.2009 19749158 PMC2781369

