# Supplementary figures and images for: AT1 Receptor Mediated Hypertensive Response to Ang II in the Nucleus Tractus Solitarii of Normotensive Rats Involves NO Dependent Local GABA Release
Source: Front Pharmacol. 2019 May 3;10:460. doi: 10.3389/fphar.2019.00460 (PMC6509664; doi:10.3389/fphar.2019.00460)

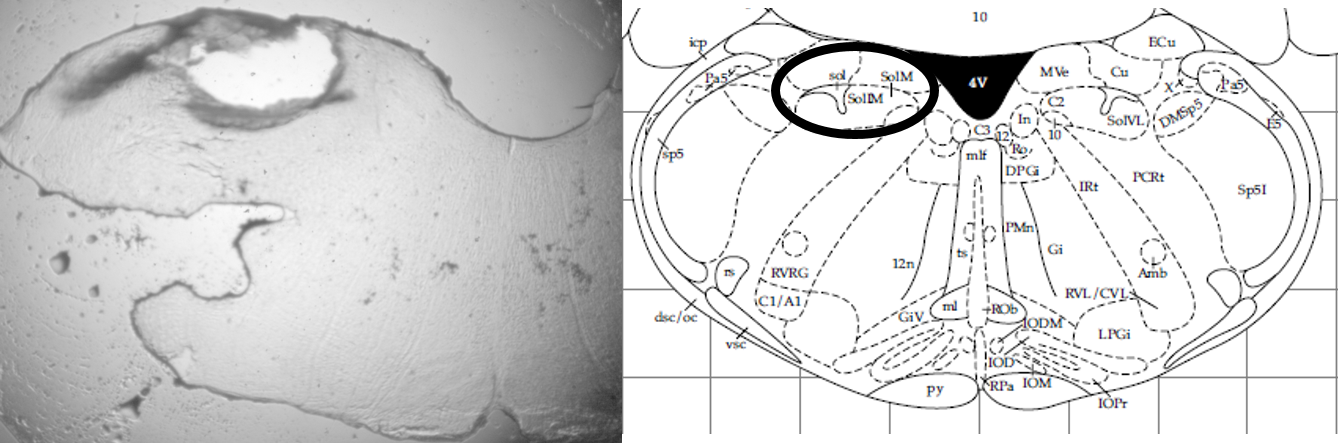

Supplement: FIGURE S1 — Histological verification of the probe localization by a neutral red staining compared against an anatomic atlas (Paxinos and Watson, 1998). [file Image_1.TIF]
